# Structural and Thermodynamic Approach to Peptide Immunogenicity

**DOI:** 10.1371/journal.pcbi.1000231

**Published:** 2008-11-21

**Authors:** Carlos J. Camacho, Yasuhiro Katsumata, Dana P. Ascherman

**Affiliations:** 1 Department of Computational Biology, University of Pittsburgh, Pittsburgh, Pennsylvania, United States of America; 2 Department of Medicine, Division of Rheumatology and Clinical Immunology, University of Pittsburgh School of Medicine, Pittsburgh, Pennsylvania, United States of America; Max-Planck-Institut für Informatik, Germany

## Abstract

In the conventional paradigm of humoral immunity, B cells recognize their cognate antigen target in its native form. However, it is well known that relatively unstable peptides bearing only partial structural resemblance to the native protein can trigger antibodies recognizing higher-order structures found in the native protein. On the basis of sound thermodynamic principles, this work reveals that stability of immunogenic proteinlike motifs is a critical parameter rationalizing the diverse humoral immune responses induced by different linear peptide epitopes. In this paradigm, peptides with a minimal amount of stability (ΔG_X_<0 kcal/mol) around a proteinlike motif (X) are capable of inducing antibodies with similar affinity for both peptide and native protein, more weakly stable peptides (ΔG_X_>0 kcal/mol) trigger antibodies recognizing full protein but not peptide, and unstable peptides (ΔG_X_>8 kcal/mol) fail to generate antibodies against either peptide or protein. Immunization experiments involving peptides derived from the autoantigen histidyl-tRNA synthetase verify that selected peptides with varying relative stabilities predicted by molecular dynamics simulations induce antibody responses consistent with this theory. Collectively, these studies provide insight pertinent to the structural basis of immunogenicity and, at the same time, validate this form of thermodynamic and molecular modeling as an approach to probe the development/evolution of humoral immune responses.

## Author Summary

In the current paradigm of immune system recognition, T cell receptors bind to relatively short peptide sequences complexed with major histocompatibility complex proteins on the surface of antigen presenting cells, while B cell receptors recognize unprocessed protein structures. Yet, ample data exist showing that peptide immunization can trigger B cell responses targeting both the immunizing peptide and peptidelike motifs contained within intact protein—despite the fact that the folding stability of such peptides is often quite low. Using thermodynamic modeling and the technique of molecular dynamics simulations, this work provides a cogent framework for understanding the relative capacity of inherently unstable peptide structures to faithfully trigger B cell antibody production against specific conformational motifs found in native/intact proteins.

## Introduction

In the conventional paradigm of humoral immune responses, B cells recognize conformational epitopes of protein antigens through interactions with surface expressed immunoglobulin receptors [1]. For most antigens, this process requires T cell help that results in sequential steps of class switching, affinity maturation, and epitope spreading [2]–[5]. The nature of the antigen itself influences this highly orchestrated process, as glycosylation patterns and other post-translational protein modifications often impact the affinity and specificity of the immunoglobulin binding domain for relevant three-dimensional epitopes [6]–[9].

Based on this mechanism of B cell activation and immunoglobulin production, native protein should be highly immunogenic relative to short peptide sequences less than 20 amino acids in length. While this concept may hold true for many antigens, the existing literature does provide examples of peptides capable of stimulating antibody production not only against the immunizing peptide, but also against corresponding regions of the native protein [10],[11]. This apparent contradiction is often resolved by assuming that peptides are capable of adopting stable structures mimicking those found in the native protein [12]–[15]. In particular, Gros and collaborators [16] have shown that the stability of synthetic, cyclized peptides mimicking an immunodominant loop of the *Neisseria meningitidis* protein PorA correlates with immunogenicity. However, because typical linear peptides are inherently unstable, with stabilities that are virtually impossible to assess due to the lack of a well defined folded (reference) state, more complete elucidation of the molecular mechanism(s) underlying these empirical observations remains elusive. Underscoring the complexity of this problem, an analysis involving a helical motif of the enzyme barnase represents the only published measurement of peptide folding free energy (ΔG_f_ = −1 kcal/mol) [17].

In the current study, we have reexamined this issue through detailed analysis of serologic profiles generated in mice immunized with overlapping 18 amino acid peptides comprising the amino terminal portion of histidyl-tRNA synthetase (HRS = Jo-1), an autoantigen implicated in the pathogenesis of idiopathic inflammatory myopathy and the anti-synthetase syndrome [18]. Our published murine model of this disease demonstrates that many of these peptides are highly immunogenic, inducing antibodies that cross react with recombinant murine HRS protein in a predictable, species-specific manner [19].

Beyond the definition of immunodominant peptides dictating B cell recognition of HRS peptide/protein combinations, this analysis has permitted correlation of the humoral immune response with structural and thermodynamic determinants of peptide immunogenicity. Of note, molecular modeling calculations indicate that although peptides are intrinsically disordered and therefore less stable than full protein, they are capable of adopting relevant structural “mimetopes” with enough stability to trigger humoral responses against corresponding regions of native protein. Immunization experiments verify that selected peptides predicted to form higher order structures similar to those existing in parent proteins induce significant antibody responses against intact protein. Moreover, competition experiments show that several of these immunogenic peptides are able to bind to stimulated antibodies with similar affinity to that of the full protein. Collectively, these studies provide insight pertinent to the structural basis of immunogenicity and, at the same time, validate this form of thermodynamic and molecular modeling as a tool to probe the development/evolution of humoral immune responses.

## Results

### Thermodynamic Relationship between Peptide Stability and Antigenicity

**Figure 1:**
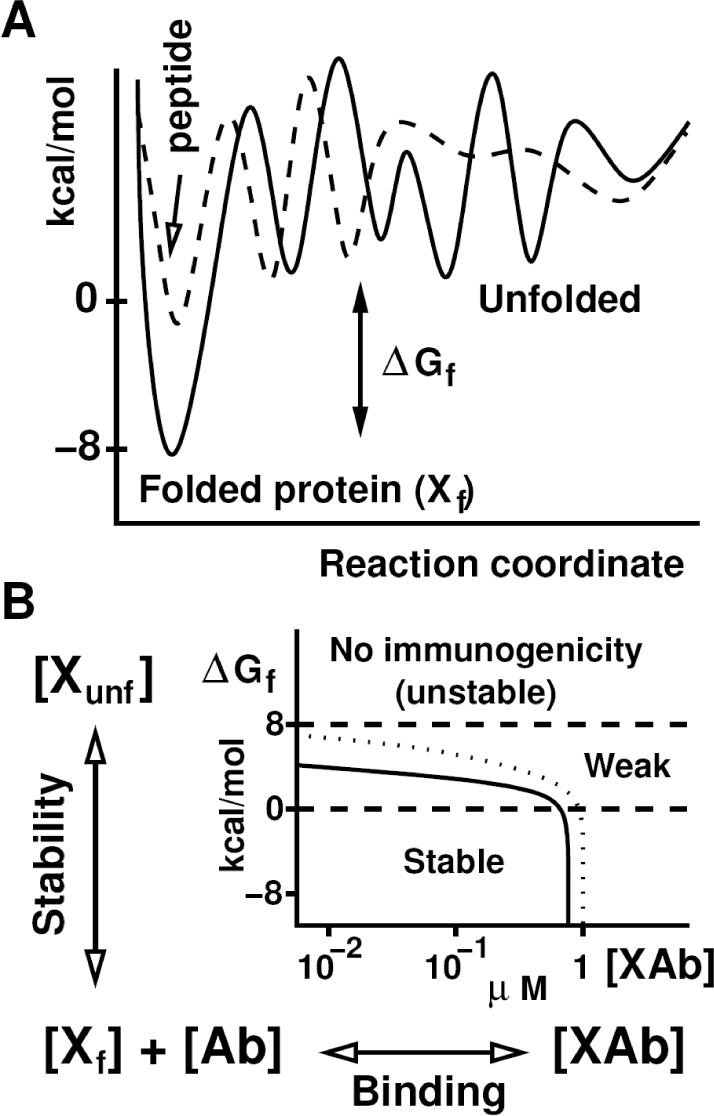
Thermodynamics and binding kinetics of a stable protein compared to weakly stable peptides sharing the same binding motifs. (A) This sketch represents the folding free energy landscape of a stable protein (solid line) relative to a less stable protein/peptide (dashed line) sharing the same folded motif. (B) Coupling of protein folding free energy and protein (X)-antibody (Ab) binding kinetics is shown in the designated graph where the amount of protein-antibody complex (XAb) complex formed is plotted as a function of the folding stability of X, with fixed concentrations of X and Ab equal to 1 µM. The solid line reflects association and dissociation rates of 10^6^ M^−1^s^−1^ and 10^−1^ s^−1^ (K_d_ = 100 nM), respectively, whereas the dotted line indicates a dissociation rate of 10^−3^ s^−1^ (K_d_ = 1 nM). Dashed lines delineate different binding regimes as a function of peptide/protein stability, identifying a “stable” category with ΔG_X_<0 kcal/mol that corresponds to peptides capable of binding to antibody with the same affinity as protein. Above this threshold exist “weakly stable” peptides that can trigger anti-protein antibodies but are unable to compete with protein for antibody binding. Peptides exceeding the folding free energy boundary of the latter category are unstable and therefore non-immunogenic.

To establish a thermodynamic basis for previous observations linking peptide immunization with humoral immune responses against native protein structural motifs, we examined the relationship between peptide folding stability and antibody-antigen binding. Although the capacity of intrinsically disordered peptides to generate and effectively bind antibodies recognizing three-dimensional epitopes appears counterintuitive, the kinetic scheme in Figure 1 (equations are in Figure S1A) demonstrate that, under very general conditions, complete peptide stability is not a necessary condition for effective binding. Indeed, classification of peptides according to the free energy (ΔG_X_) of their protein-like motifs defines three classes of peptides possessing very different immunogenic properties. These categories include: (a) “stable” peptides (for which ΔG_X_<0 kcal/mol) that can form the same number of peptide-antibody ([XAb]) complexes as stable protein despite a wide range of folding free energy values; (b) “weakly-stable” peptides with ΔG_X_>0 kcal/mol (but <8 kcal/mol) that have a drastic decrease in antibody binding events relative to the full protein; and, (c) “unstable” or “non-immunogenic” peptides with ΔG_X_>8 kcal/mol and resulting unfolding rates of 10^9^ s^−1^ or higher that preclude any effective binding [20],[21]. While the precise stability thresholds are somewhat dependent on concentration and binding affinities, the relative stability grouping of each peptide type is independent of folding rates.

### Antibody Profiles Generated through HRS Peptide Immunization

**Figure 2:**
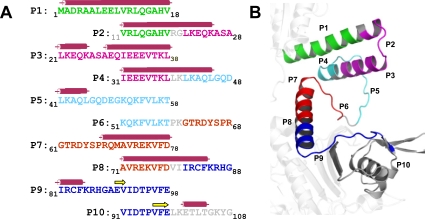
Linear sequence and structural model of murine HRS. Panel (A) depicts the linear sequence of overlapping 18 amino acid peptides comprising the amino terminal 108 amino acids of murine HRS. Color-coding corresponds to the composite three-dimensional model shown in panel (B) (derived from the structures of human and *Thermoplasma acidophilum* HRS as described in Methods), demonstrating the relationship of these sequences to various structural motifs. Sequences extending beyond peptide 10 are colored in gray (amino acids 108–151) or semi-transparent white (amino acids 152–510). Bars overlying the amino acid sequence in panel A signify α-helices, arrows indicate β-sheets, and underlining identifies proline residues.

As an example of the epitope classification scheme derived from this thermodynamic analysis, we have mapped relevant B cell epitopes of histidyl-tRNA synthetase (HRS) through peptide immunization of NOD.*Idd3/5* mice. As shown in Figure 2, the panel of HRS peptides consists of overlapping 18 amino acid sequences corresponding to the immunodominant amino terminal portion of HRS. The relationship between these peptides and different structural motifs of intact protein is highlighted by the accompanying model of HRS.

**Figure 3:**
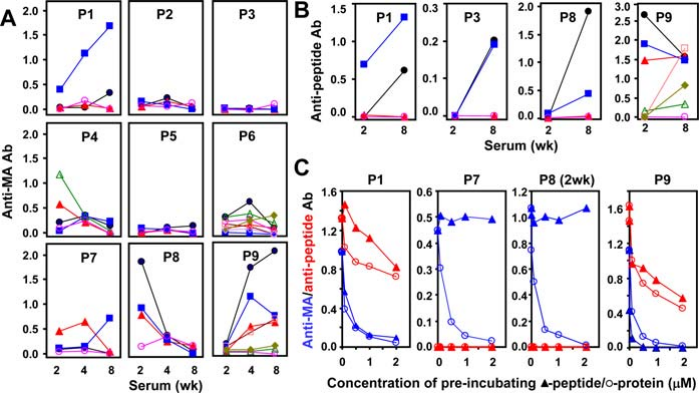
Temporal evolution of HRS peptide-induced antibody responses and characterization of relative peptide vs. protein binding affinity. Symbols denote data points corresponding to the mean OD_450_ values of triplicate ELISA samples (after subtraction of no antigen and anti-MBP background) generated by sera obtained from individual peptide-immunized mice, and lines serve as a visual guide. Individual panels in A) depict levels of peptide-induced antibodies recognizing a HRS fusion protein (consisting of the amino terminal 151 amino acids of murine HRS linked to maltose binding protein = MA/MBP) at various time points after a single immunization with peptide/CFA emulsions. The plots in B) show corresponding anti-peptide antibody titers induced by immunization with the indicated peptides (line colors identify matched serum samples). Error bars are negligible and have therefore been omitted. Finally, panel C) demonstrates competition assays in which serum obtained from HRS peptide-immunized mice (8 weeks following immunization unless otherwise indicated) is pre-incubated with different molar concentrations of immunizing peptide (closed triangles) or MA/MBP (open circles) before being subjected to ELISA. Substrate antigens consist of the immunizing peptide (red lines and symbols) or MA/MBP (blue lines and symbols). Molar concentrations of peptide and protein used for pre-incubation are equivalent at individual points along the x-axis. OD_450_ values again represent the mean of triplicate samples; negligible error bars are not shown.

Review of Figure 3A indicates that several peptides comprising the amino terminal 98 amino acids of HRS generate antibody responses against a HRS fusion *protein* (MA/MBP = amino terminal amino acids 1–151 linked to maltose binding protein) by two weeks, most notably peptides 1 (a.a. 1–18), 4 (a.a. 31–48), 6 (a.a. 51–68), 7 (a.a. 61–78), 8 (a.a. 71–88), and 9 (a.a. 81–98). Temporal assessment of anti-HRS protein antibody responses induced by these peptides and comparison to antibody responses against the immunizing peptide (Figure 3B) demonstrates several different recognition patterns consistent with the thermodynamically-defined categories in Figure 1. In the case of peptides 1 and 9, for example, titers of anti-HRS protein and anti-peptide antibodies parallel each other by tending to increase over time. Conversely, peptides 4, 6, and 7 produce more variable temporal patterns of anti-HRS protein antibody responses, generally without corresponding anti-peptide responses over the monitored time course (significant anti-P6 titers develop in only 1/8 P6 immunized-mice at 8 weeks). Finally, peptides 2 and 5 represent sequences that fail to generate anti-protein or anti-peptide antibodies at any time point.

### Competition ELISAs Reflect Structural Stability of HRS Peptides

Complementing these results, competition ELISAs provide further insight regarding the relative antigenicity of HRS peptides and protein. As shown in Figure 3C, pre-incubating sera from peptide-immunized mice with increasing concentrations of MA/MBP effectively reduces residual binding to MA/MBP substrate, confirming the specificity of antibody responses generated by peptides 1, 7, 8, and 9. However, when peptides are used in the pre-incubation phase, the effect is more variable. With peptide 7- and 8-immunized sera, for example, peptide pre-incubation has little or no detectable effect on the ability of antibodies to bind MA/MBP. On the other hand, molar equivalent amounts of peptide 1 and 9 compete for antibody binding to both MA/MBP and peptide substrate as effectively as protein—consistent with the ability of peptides 1 and 9 to adopt relatively stable structures in solution that resemble corresponding regions of intact protein.

### Molecular Dynamics (MD) Simulations Provide Measure of Structural Stability of Proteinlike Motifs

To correlate these peptide immunization studies with the thermodynamically-defined categories of immunogenicity outlined in Figure 1, we employ MD simulations. However, the inherent difficulty in directly measuring peptide folding free energy is also present in MD–namely, the “folded” state of interest (i.e., the motif that binds the pool of B cell receptors) is not well defined. A second drawback is that an absolute thermodynamic estimate of free energy needs to account for the unstructured, unfolded state. Cutting edge MD techniques can compute free energy differences between well defined states and may be able to account for the configurational entropy of peptides, but currently cannot properly estimate the required entropy of ∼7000 explicit water molecules [22].

Despite this caveat, the dashed line in Figure 1A indicates that the stability of states other than the protein-like motif (X) is irrelevant from the point of view of establishing a correlation between antibody binding of stable protein versus unstable linear peptides. Other states could, of course, lead to an immune response targeting an unknown structure. We note, however, that this scenario does not apply here, since ELISAs involving peptide substrates do not seem to yield a signal if there is no response against protein. The only exceptions are motifs represented by peptides 3 and 8 which, as argued below, are obscured in their protein form. Hence, MD simulations represent a valuable and insightful alternative method for probing the relative stability of different epitopes in their corresponding protein fold and for better defining the relevant “folded” state. In particular, because recognition events occur within a nanosecond time scale [21],[23], peptides are simulated over a 10 nanosecond period [16] that allows extraction of the most stable backbone protein-like motifs of four consecutive amino acids (i.e., a small binding domain).

**Figure 4:**
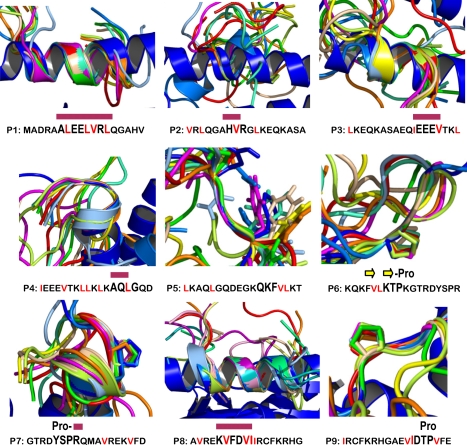
Molecular dynamics simulations of HRS-derived peptides. Three-dimensional conformations of individual 18 amino acid HRS peptides (presented in Figure 1) were simulated for 10 nanoseconds. Each simulation consists of 10 snapshots (from light blue to red) separated by 1 nanosecond intervals and superimposed on a structure matching the corresponding motif present in full protein (shown here in blue, but also depicted in Figure 1). Peptide sequence is again shown below respective simulations, with hydrophobic residues in red and conserved regions in a larger font. Structural motifs for each conserved region are designated by the following symbols: red bars = helices, yellow arrows = β-sheets, and Pro = proline residues. For peptide 5 (P5), Gln_12_ and Phe_14_ are shown as sticks (in the figure) to indicate that the conserved backbone is blocked by flanking side chains.

**Figure 5:**
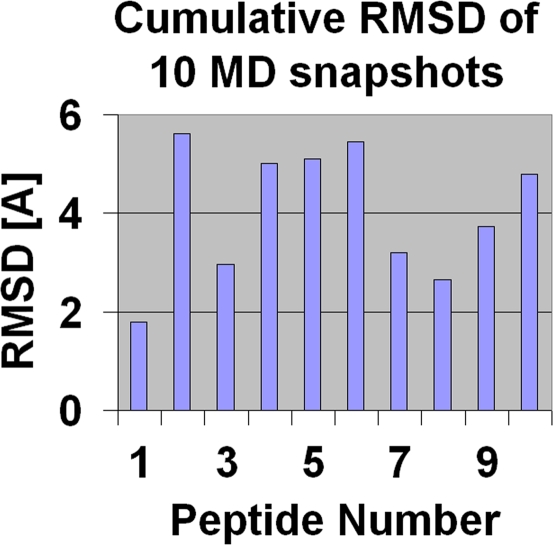
Cumulative root-mean-squared-deviation (RMSD) of optimal structural alignments of proteinlike motifs represented in linear peptides. Identification of 4 consecutive amino acid stretches yielding the minimum cumulative pairwise root-mean-squared-deviation (RMSD) for snapshots included in the 10 nanosecond period forms the basis of optimal structural alignment for individual peptides. Lower values indicate more stable peptides with less structural variation.

Figure 4 shows optimal backbone structural alignments of MD snapshots superimposed on the three-dimensional model of murine HRS. The alignment for each peptide is based on the 4 consecutive residues with the smallest cumulative root-mean-square deviation (RMSD) over a 10 nanosecond period [16] (summarized by the bar graph in Figure 5). Although peptide conformations fluctuate to varying degrees, *the composite profiles of the most structurally stable protein-like motifs provide a visual analogue showing relative stability and similarity to defined motifs found in murine HRS*. Interestingly, each of the peptides with a cumulative RMSD value less than 4 Å (i.e., peptides 1, 3, 7, 8, and 9) triggers affinity maturation towards MA/MBP and/or peptide, whereas peptides with less stable backbone structures (peptides 2, 4, 5, and 6) typically do not promote this temporal pattern of increasing antibody titer. Of note, MD simulations indicate that for those peptides capable of adopting higher order structure, the identified motifs can persist for several nanoseconds—a time period sufficient for antibody recognition [21],[23].

A more detailed analysis of the hydrogen bond (HB) networks [24] sampled during the MD runs yields similar conclusions, with the caveat that proline-stabilized structures such as peptide 9 do not involve HBs. Stable HBs from motifs both present and missing in the native protein (Figure 2) are listed in Table 1. Consistent with the RMSD results, peptides 1, 3, and 8 preserve protein-like motifs that involve several HBs for a significant amount of the simulation time. Peptide 7 also preserves a HB at the beginning of a helix that, together with Pro7, contributes to stability of the motif. Peptide 4 has one stable HB at the end of a helix (no proline), providing a degree of structural stability that is consistent with the ability of this peptide to generate an initial antibody response against protein two weeks following immunization (Figure 3A). Despite the fact that peptides 2 and 5 have some secondary structure, these peptides do not preserve their corresponding HBs and fail to trigger antibodies against protein or peptide.

**Table 1:** Proteinlike and peptide-unique motifs stabilized by hydrogen bonds (HBs) (error 10%).

Peptide Number	HBs Present in Protein	Average Stability of HBs	HBs Not in Protein	Average Stability of HBs	Comment
1	6–14: **5** HBs helix	89%	-	-	Stable helix
2	8–12: **1** HB helix	29%	12–14 bb-bb	41%	Unstable peptide
3	11–17: **3** HBs helix	52%	6–13 bb-sc	24%	3 helix bonds, but sc of conserved motif E_12_E_13_E_14_ are forming intramolecular bonds
4	12–16: **1** HB helix	54%	12–18 bb-sc	66%	Last helix turn is stable
5	1–5: **1** HB helix	27%	2–9 bb-sc	44%	Unstable peptide
6	4–6 bb-bb (?)	43%	-	-	Peptide is not resolved experimentally
7	6–10: **1** HB helix	78%	7–12 bb-bb	56%	First helix turn is stabilized by HB and Pro
8	4–11: **4** HBs helix	75%	6–11 bb-bb	51%	Stable helix
9	-	-	3–17 bb-bb	56%	Stable Pro-peptide; HB 3–17 stabilizes the presentation of the Pro-motif (12–14)
10	9–13: **1** HB helix	22%	8–11 bb-bb	27%	Unstable peptide, Pro-motif is more unstable than in peptide 9

bb: backbone; sc: side chain.

Coupled with the thermodynamic modeling of Figure 1, these findings strongly suggest that the highly immunogenic peptides 1 and 9 fall into the stable category where ΔG_X_ values allow maximal peptide-antibody complex formation. In contrast, this combined analysis indicates that peptides 4 and 6 are weakly stable, with ΔG_X_ values that favor diminished antibody binding of peptide relative to full protein. This classification is fully consistent with ELISAs (Figure 3) showing that antibodies generated by immunization with peptides 4 and 6 generally bind protein, but not peptide, substrate antigens. Also dovetailing with experimental results, the non-immunogenic peptides 2 and 5 lack any form of structure resembling native HRS (Table 1), and no new structural motifs are detected within the limited simulation time. The latter observation also reflects the fact that although MD simulations and resulting RMSD calculations based on backbone stability provide a framework for ranking the likelihood of forming high affinity peptide-antibody complexes, side chains remain a critical determinant influencing the specificity of this interaction [25]. More specifically, the loop structure of peptide 5 (shown in Figure 4) is flanked by highly unstable side chains blocking the relatively conserved backbone. With peptide 3, on the other hand, intramolecular HBs linking side chains of Ser_7_ and Gln_10_ to side chains of the structurally conserved motif E_12_E_13_E_14_ (44% and 29%, respectively) might be responsible for the weak anti-peptide response shown in Figure 3B.

## Discussion

Collectively, these studies show that several peptides corresponding to the amino terminal portion of murine HRS are capable of inducing anti-protein antibodies of varying affinity and temporal persistence. As shown by molecular dynamics simulations, sequences of the most immunogenic peptides correspond to highly ordered structural motifs in the parent protein. Competitive ELISAs provide direct evidence that these peptides share structural determinants with native protein by demonstrating the relative equivalence of antibody affinity for HRS protein (MA/MBP) and selected peptides (i.e., antibodies recognize or identify, rather than actively define, the immunodominant motif). Of greater significance, first principle calculations and molecular dynamics simulations underscore the thermodynamic and structural basis of these experimental observations.

Among the most interesting findings emerging from the experiments summarized in Figure 3 is the diversity of antibody responses engendered by immunization with different peptides. While peptides 1 and 9, for example, bind induced antibodies almost as effectively as full protein, peptides 4 and 7 generate strong antibody responses to protein that fail to recognize peptide in the context of ELISA. In contrast, peptides 2 and 5 do not support antibody production against either protein or peptide. For those peptides generating strong antibody responses against the HRS fusion protein MA/MBP, structural mapping indicates correspondence to well-defined domains that involve either α-helices (peptides 1, 3, 4, 7, 8) or linear motifs stabilized by a proline residue (peptides 6, 7, 9). To some extent, this result is expected because (in solution) such motifs should retain some of the stability present in native protein. The key question, however, is how peptides bearing only partial structural resemblance to native protein can bind antibodies with similar affinity to that of intact protein.

Answering this question relies on the simple observation that although peptides should be destabilized when isolated from protein (e.g., due to solvent exposure of normally buried amino acid residues), this instability does not translate into an equivalent drop in affinity towards the repertoire of B cells receptors. Indeed, thermodynamic calculations in Figure 1 reveal a relatively broad range of ΔG_f_ values (<0 kcal/mol) in which peptides are capable of triggering an immune response similar to full protein. Hence, as long as the peptide fold resembles that of the full protein, this class of peptides (defined as “stable” in Figure 1) should have antigenic properties similar to those of full protein. Beyond those “stable” peptides with ΔG_f_<0 kcal/mol, Figure 1 identifies a “weakly-stable” regime where peptides are typically 10–100 times less likely than HRS protein to bind peptide-induced antibodies. In other words, the same antibodies that rarely bind isolated peptides can readily recognize the corresponding motif in the context of stable protein. Unlike their more stable counterparts, however, such weakly stable motifs typically do not promote affinity maturation against protein (compare peptides 1 and 9 to peptides 4 and 6, Figure 3).

From a modeling point of view, molecular dynamics (MD) simulation in explicit solvent represents the most accurate approach to assess peptide stability. Although this technique has time limitations that prevent a full thermodynamic analysis of each peptide, the 10 nanosecond period used here is sufficient to assess the stability of protein-like conformations relevant to the comparison of peptide- versus protein-targeted antibody responses. Clearly, the MD simulations demonstrate a wide range of structural stabilities over 10 nanosecond runs; in the case of peptides 1 and 9, however, the composite structural motifs greatly resemble those presented by full protein, confirming that helical as well as some proline-based linear motifs can preserve their structural integrity over a time frame that is fully compatible with molecular recognition [21],[23].

Perhaps the differences in stability and antibody binding affinity between overlapping sequences of peptide 9 (amino acids 81–98) and peptide 10 (amino acids 91–108) best illustrate the power as well as predictive potential of MD simulation. While competition ELISAs demonstrate that sera derived from peptide 9-immunized mice recognize both peptide 10 and peptide 9 (consistent with the immunodominant proline-containing epitope suggested by MD that encompasses amino acids 93–96), the relative affinity for peptide 9 exceeds that for peptide 10 by a log order of magnitude (data not shown)—a result that again correlates with MD simulations showing that the same proline-containing motif is significantly destabilized by surrounding sequence in peptide 10, but not in peptide 9 (see RMSD analysis, Figure 5 and Table 1).

Based on the overall molecular dynamics analysis performed in this study, peptides 1, 3, 7, 8, and 9 best preserve the folded structure found in corresponding regions of native protein. This finding is consistent with the data in Figure 3 showing that each of these peptides induces some degree of affinity maturation against either peptide or MA/MBP protein. With some peptides, however, the failure to stimulate antibodies increasingly cross-reactive with their corresponding HRS structural motifs appears to conflict with the MD stability predictions. For example, peptide 3 shows no anti-MA/MBP response at any time point. Yet, analysis of the HRS structure in Figure 2 suggests that peptide 3 is sterically hindered by one side of the α-helical motif of peptide 8, resulting in mutual epitope blockade. Note that the suggested negatively charged tri-glutamate epitope of peptide 3 is predicted to face at least four positively charged groups from peptide 8, further promoting such blockade (see Figure 4 for additional structural detail). Interestingly, the MA/MBP construct (Figure 2) still leaves one side of the helix of peptide 8 (i.e., the hydrophobic side) exposed, suggesting that the anti-MA/MBP and anti-peptide responses generated by this peptide (Figure 3) might be against different faces of this structural motif.

Beyond these structural considerations pertinent to peptides 3 and 8, the relatively indiscriminate 2 week antibody responses shown in Figure 3 support the prevailing view that early humoral activation involves a lower binding specificity threshold [26]–[29] than that required for affinity maturation. The more novel thermodynamic counterpart of this observation is shown in Figure 1, where 100-fold differences in binding affinity have little effect on the formation of antigen-B cell receptor (BCR) complexes involving stable peptides. Even with weakly stable peptides (e.g., peptides 4 and 6) where the impact of binding affinity is potentially more significant, early antibody responses *against protein* can occur—often with titers that are indistinguishable from those generated by their more stable counterparts. In fact, from the standpoint of stability, Figure 1 suggests that peptides need only eclipse the free energy threshold separating unstable from stable/weakly stable peptides to support early antibody formation.

In contrast, the stability threshold differentiating stable and weakly stable peptides appears to play a greater role in determining those peptides capable of generating long term antibody responses, likely reflecting a requirement for sustained antigen-BCR interactions. Perhaps peptide 4 best illustrates the immunogenic relevance of this interplay between binding specificity and stability thresholds. A weakly stable peptide (see Figure 4) that is also the most hydrophobic of all the assessed HRS peptides, peptide 4 triggers unusually high antibody titers at week 2; however, none of these initial responses overcomes the higher activation threshold required to induce affinity maturation. Although additional factors modulate the selection process that leads to progression/maturation of the humoral immune response, the evidence presented here indicates that this more stringent activation threshold is intimately related to peptide structural stability.

Complementing the overall experimental evidence of HRS peptide immunogenicity presented in these studies, the literature is replete with examples of peptide immunization leading to antibody responses against parent protein (reviewed in references [10],[11]). While the original studies involving these peptides do not invoke the novel thermodynamic computation and molecular dynamics simulations employed in this work, complementary analysis indicates that several of the reported peptides are capable of forming higher order structures such as α-helices and proline-stabilized domains. Moreover, preliminary application of our theoretical and quantitative framework to alternative peptide antigens has yielded data (not shown) consistent with these findings and again demonstrates the power/versatility of this approach in characterizing epitope recognition. However, what is most remarkable about the thermodynamic classification scheme outlined in this work is that peptides with an extraordinarily wide range of folding free energies (but with structurally conserved core motifs) behave as “stable” peptides capable of triggering an immune response against defined motifs present in full protein.

Given such links to the immunobiology of antibody-antigen recognition, this work suggests a number of important experimental applications involving the described thermodynamic modeling/computational analysis. First, more precise mapping of B cell responses over time will help define the sequence of molecular recognition events leading to epitope spreading and, in the process, elucidate the structural component of this process that clearly involves additional factors such as side chain conformation, relative hydrophobicity/hydrophilicity, and overall epitope accessibility (steric freedom). Second, identification of immunodominant peptide epitopes will permit more detailed categorization of disease subsets and correlation with disease activity. Finally, this computational tool will facilitate the prediction and design of immunodominant peptide epitopes that can be used to define novel autoantibody specificities in patients with underlying autoimmune diseases. Through such identification of autoantigen panels, this approach may provide insight regarding more general epigenetic shifts that generate multiple autoantigens and ultimately lead to autoimmunity.

## Materials and Methods

### Antigen Preparation

Overlapping peptides (18–20 mers) comprising the amino terminal 108 amino acids of murine histidyl-tRNA synthetase (HRS) were synthesized and HPLC purified by the University of Pittsburgh Molecular Medicine Institute using Fmoc chemistry. As previously described, recombinant murine HRS was generated as a maltose binding protein (MBP) fusion protein following subcloning of the appropriate sequence (derived from RT-PCR amplification of C57BL/6 myocyte RNA) into the bacterial expression vector pMALc2 (New England Biolabs, Ipswich, MA) [19]. *In situ* mutagenesis (Stratagene, La Jolla, CA) with insertion of a stop codon after base pair 453 yielded a construct encoding the amino terminal 151 amino acids of murine HRS fused to MBP (MA/MBP). Expressed proteins were purified with amylose resin per the manufacturer's protocol (New England Biolabs, Ipswich, MA), filter sterilized, and then subjected to additional column purification for endotoxin removal (Profos AG, Regensburg, Germany) prior to use in ELISAs.

### Mouse Immunization

NOD.*Idd3/5* (C57BL/6 Insulin dependent diabetes *Idd3/5* non-MHC loci transgressed onto the NOD background) mice were bred in our animal facility. Eight to ten week old mice were used in immunization protocols approved by the University of Pittsburgh IACUC. PBS containing 90 µg of the indicated peptides was emulsified with CFA in a 1∶1 ratio and then injected at the base of the tail in a total volume of 200 µl. Pertussis toxin (Sigma-Aldrich, St. Louis, MO) was administered intraperitoneally (200 ng/mouse in 100 µl PBS) at the time of immunization and 48 hours later. Mice were tail-bled 2 and 4 weeks after immunization. 8 weeks post immunization, mice were sacrificed, and additional blood was collected from the heart.

### ELISA for Serum Anti-Protein and Anti-Peptide Antibodies

Standard solid phase ELISAs provided measurements of IgG anti-MA/MBP and anti-HRS peptide antibody levels in the sera of mice immunized with different HRS peptides [19]. Briefly, appropriately diluted serum samples (1∶500) from immunized mice were added to wells containing substrate antigens that included MA/MBP (2 µg/ml), MBP (2 µg/ml), HRS peptide (2 µg/ml), or no antigen. Following a 60 minute incubation with horseradish peroxidase-conjugated goat anti-mouse IgG (0.04 µg/ml, Santa Cruz Biotechnology, Santa Cruz, CA), enzymatic reactions were visualized using 3,3,5,5-Tetramethylbenzidine (TMB) (Sigma-Aldrich) and subsequently terminated with 1 N H_2_SO4. Color development was measured at 450 nm by a Wallac 1420 multilabel counter (PerkinElmer, Wellesley, MA), and values were plotted as OD_450_ substrate antigen - OD_450_ no antigen. All assays were performed in triplicate wells.

### Competitive ELISA for Serum Anti-Protein and Anti-Peptide Antibodies

Plates were coated and blocked as described above. Diluted serum samples (1∶250) were mixed in a 1∶1 ratio with serially diluted MA/MBP or HRS peptide solutions in microtubes and preincubated for 30 minutes at room temperature. Preincubated samples (final serum dilution of 1∶500) were then applied to the plates and incubated for another 2 hours at room temperature. ELISAs were completed using the same protocol as described above.

### Structural Modeling of Murine Histidyl-tRNA Synthetase

The structural model of *Mus musculus* histidyl-tRNA synthetase (HRS = Jo-1) in Figure 1 concatenates the NMR structure of the Whep-Trs domain (Protein Data Bank-PDB code 1X59, unpublished) of human HRS (amino acids 1–64) and a homology model of residues 60–498 that is based on the crystal structure of *Thermoplasma acidophilum* HRS (PDB code 1WU7, unpublished). With more than 25% sequence identity, including perfect matching of prolines and glycines in the domains of peptides 1 to 9 listed in Figure 1, the alignment shown in Figure S1B and the corresponding homology model represent a robust working model of the full protein [25],[26]. Only the linker region encompassing residues 46 to 68 (represented by peptide 6 (amino acids 51–68) in Figure 2) is not well resolved in either the NMR or the crystal structures.

### Thermodynamic and Kinetic Modeling of Peptide Stability

Figure 1 solves the standard rate equations for folding and binding of protein/peptide based on typical thermodynamic parameters and the assumption that protein (X) binds antibody (Ab) only when folded in state X_f_. The results in Figure 1B depend only in the folding free energy (independent of the folding rates), which is varied to cover the full range between −8 and 8 kcal/mol. Under the additional assumptions that appropriately folded peptides fully encompass the corresponding protein binding domain and that antibody-antigen association and dissociation rates are 10^6^ M^−1^s^−1^ and 10^−1^ s^−1^ (alternative dissociation rate of 10^−3^ s^−1^ is shown as a dotted line), respectively [30], binding affinity depends more directly on the concentration of X_f_ ([X_f_]) than on peptide stability. For simplicity, we assume a concentration of antibody ([Ab]) and protein ([X]) equal to 1 µM. However, the overall shape of the curve does not change significantly with a higher or lower [Ab]. For [Ab]>1 µM, the maximum amount of complex [XAb] remains the same, but the stability thresholds (dashed lines in Figure 1) move up. For [Ab]<1 µM, the amount of complex will be limited by [Ab], and the stability threshold will decrease only slightly.

### Molecular Dynamics Simulations

Molecular dynamics simulations were performed using the MD simulation package GROMACS 3.3.1 [31] on individual peptides of HRS. Each peptide was centered in a rhombic dodecahedron box with a 15 Å minimum distance from the protein surface to the box edges. The resulting system was solvated with simple point charge water molecules and then minimized by using steepest descent method with the GROMOS96 force field. Counter ions were added to neutralize the system. The temperature was coupled to a bath of 300K with a coupling time constant of 0.1 ps. The pressure was coupled to 1 Bar using a 0.5 ps time constant and water compressibility of 4.5×10^−5^ Bar^−1^. A cut-off radius of 10 Å was used in the simulations for non-bonded interactions. Initial velocities were generated randomly from a Maxwell distribution at 300̊K. Simulations consisted of 10 nanosecond runs using the corresponding protein structure depicted in Figure 2 as a starting conformation for each peptide. Accuracy/reliability of the simulations was confirmed with duplicate runs for each peptide.

## Supporting Information

Figure S1 Kinetic of folding and binding and sequence alignment. (A) Folding of protein X and binding of X with antibody Ab. (B) Alignment of amino terminal 1–151 Histidyl tRNA synthetase from Mus musculus (MA) and Thermoplasma acidophilum (PDB code 1WU7)
(0.54 MB TIF)
