# Supplementary figures and images for: Structural and Thermodynamic Approach to Peptide Immunogenicity
Source: PLoS Comput Biol. 2008 Nov 21;4(11):e1000231. doi: 10.1371/journal.pcbi.1000231 (PMC2577884; doi:10.1371/journal.pcbi.1000231)

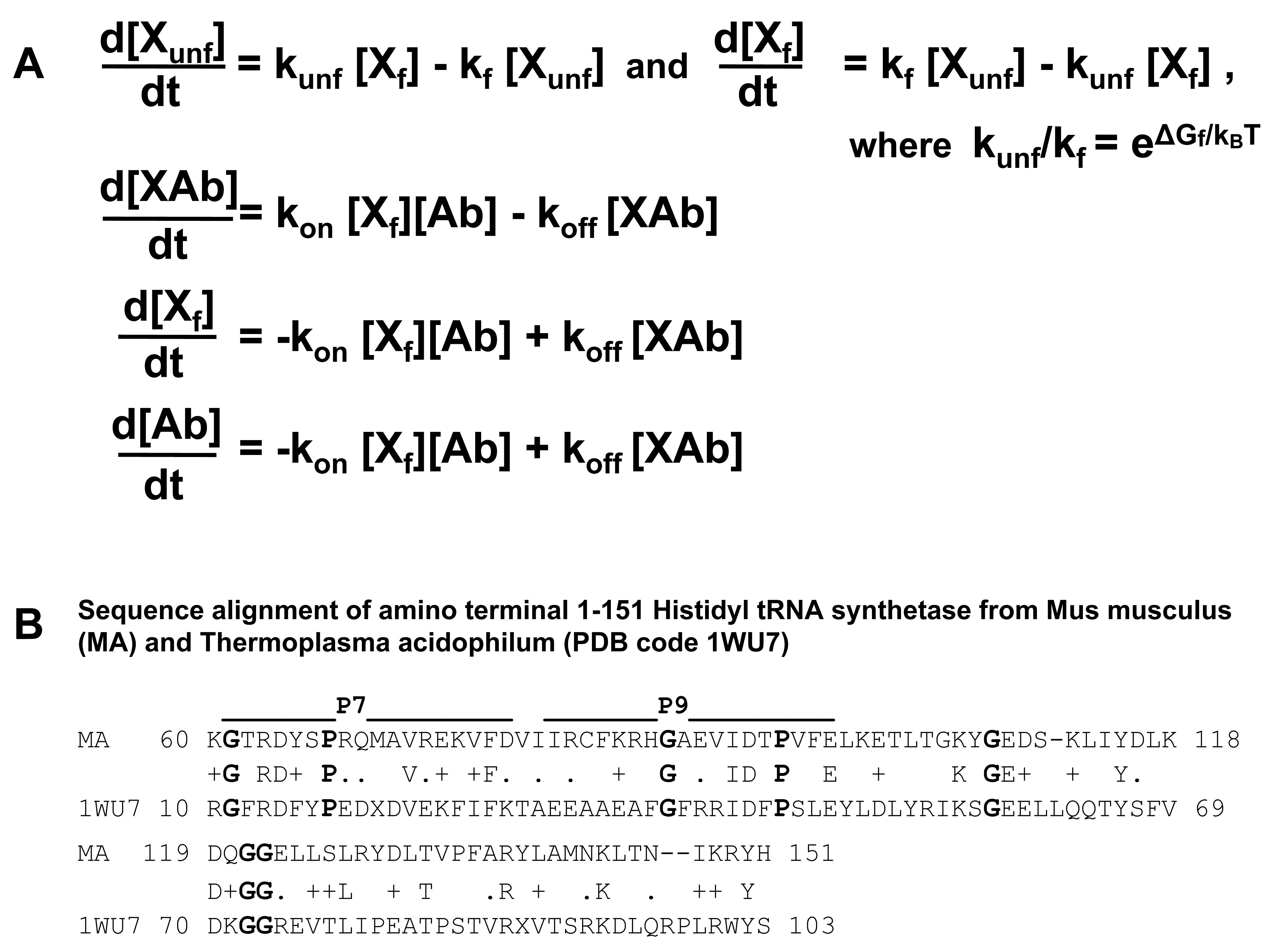

Supplement: Figure S1 — Kinetic of folding and binding and sequence alignment. (A) Folding of protein X and binding of X with antibody Ab. (B) Alignment of amino terminal 1–151 Histidyl tRNA synthetase from Mus musculus (MA) and Thermoplasma acidophilum (PDB code 1WU7) (0.54 MB TIF) [file pcbi.1000231.s001.tif]
